# Supplementation of Mangiferin to a High-Starch Diet Alleviates Hepatic Injury and Lipid Accumulation Potentially through Modulating Cholesterol Metabolism in Channel Catfish (*Ictalurus punctatus*)

**DOI:** 10.3390/antiox13060722

**Published:** 2024-06-13

**Authors:** Yutong Zheng, Qisheng Lu, Jingyue Cao, Yulong Liu, Haokun Liu, Junyan Jin, Zhimin Zhang, Yunxia Yang, Xiaoming Zhu, Dong Han, Shouqi Xie

**Affiliations:** 1 College of Fisheries and Life Science, Dalian Ocean University, Dalian 116023, China; zhengyutong@ihb.ac.cn; 2 State Key Laboratory of Freshwater Ecology and Biotechnology, Institute of Hydrobiology, Chinese Academy of Sciences, Wuhan 430072, China; luqisheng@ihb.ac.cn (Q.L.); caojingyue@ihb.ac.cn (J.C.); liuyulong@ihb.ac.cn (Y.L.); liuhaokun@ihb.ac.cn (H.L.); jinjunyan@ihb.ac.cn (J.J.); zhangzm@ihb.ac.cn (Z.Z.); yxyang@ihb.ac.cn (Y.Y.); xmzhu@ihb.ac.cn (X.Z.); sqxie@ihb.ac.cn (S.X.); 3 College of Advanced Agricultural Sciences, University of Chinese Academy of Sciences, Beijing 100049, China; 4 Hubei Hongshan Laboratory, Wuhan 430070, China; 5 The Innovative Academy of Seed Design, Chinese Academy of Sciences, Wuhan 430072, China

**Keywords:** mangiferin, starch, cholesterol metabolism, *sqle*, channel catfish

## Abstract

Starch is a common source of carbohydrates in aqua feed. High-starch diet can cause hepatic injury and lipid accumulation in fish. Mangiferin (MGF) can regulate lipid metabolism and protect the liver, but there is limited research on its effects in fish. In the present study, we investigated whether MGF could ameliorate high-starch-induced hepatic damage and lipid accumulation in channel catfish. The channel catfish (*Ictalurus punctatus*) were fed one of four experimental diets for eight weeks: a control diet (NCD), a high-starch diet (HCD), an HCD supplemented with 100 mg/kg MGF (100 MGF), and an HCD supplemented with 500 mg/kg MGF (500 MGF). The results demonstrated that the weight gain rate (WGR) (*p* = 0.031), specific growth rate (SGR) (*p* = 0.039), and feed conversion efficiency (FCE) (*p* = 0.040) of the 500 MGF group were significantly higher than those of the NCD group. MGF supplementation alleviated liver damage and improved antioxidant capacity (T-AOC) compared to those of the HCD group (*p* = 0.000). In addition, dietary MGF significantly reduced plasma glucose (GLU) (*p* = 0.000), triglyceride (TG) (*p*= 0.001), and low-density lipoprotein cholesterol (LDL) (*p* = 0.000) levels. It is noteworthy that MGF significantly reduced the plasma total cholesterol (TC) levels (*p* = 0.000) and liver TC levels (*p* = 0.005) of channel catfish. Dietary MGF improves cholesterol homeostasis by decreasing the expression of genes that are involved in cholesterol synthesis and transport (*hmgcr*, *sqle*, *srebf2*, *sp1*, and *ldlr*) and increasing the expression of genes that are involved in cholesterol catabolism (*cyp7a1*). Among them, the largest fold decrease in squalene epoxidase (sqle) expression levels was observed in the 100 MGF or 500 MGF groups compared with the HCD group, with a significant decrease of 3.64-fold or 2.20-fold (*p* = 0.008). And the 100 MGF or 500 MGF group had significantly decreased (by 1.67-fold or 1.94-fold) Sqle protein levels compared to those of the HCD group (*p* = 0.000). In primary channel catfish hepatocytes, MGF significantly down-regulated the expression of *sqle* (*p* = 0.030) and reduced cholesterol levels (*p* = 0.000). In NCTC 1469 cells, MGF significantly down-regulated the expression of sqle (*p* = 0.000) and reduced cholesterol levels (*p* = 0.024). In conclusion, MGF effectively inhibits sqle expression and reduces cholesterol accumulation. The current study shows how MGF supplementation regulates the metabolism and accumulation of cholesterol in channel catfish, providing a theoretical basis for the use of MGF as a dietary supplement in aquaculture.

## 1. Introduction

Carbohydrate, a cost-effective energy source in aquafeeds, can be incorporated at the appropriate levels to optimize feed efficiency and reduce aquafeed costs [1]. Starches are excellent sources of carbohydrates in aqua feed [2]. However, the utilization of high starch by some fish is limited, and the ingestion of excessive carbohydrate can induce negative effects such as increased plasma cholesterol levels, alterations in liver tissue morphology and hepatic damage, as well as excessive hepatic lipid deposition. [3,4,5,6]. Cholesterol is important in the body, because it is an essential component of cell membranes and an important precursor to steroid hormones, bile acids, and vitamin D [7]. However, excessive cholesterol levels can exert lipotoxic effects on cells [8]. But numerous studies have shown that hepatic free cholesterol accumulation and the disruption of hepatic cholesterol homeostasis are closely related to the pathogenesis of liver damage and non-alcoholic steatohepatitis [9,10,11]. However, very limited research has been conducted on the perturbation of cholesterol metabolism leading to liver damage and hepatic lipid deposition in fish studies.

Mangiferin (MGF) is a natural polyphenol that can be found in a variety of plants worldwide, with common occurrences in the Gentianaceae family plants, Swertia genus plants, and Salacia genus plants [12]. MGF reduces the presence of cytoplasmic vacuoles, lipid droplet deposition, and hepatocyte necrosis in hepatocytes of high-fat-diet-fed mice by regulating lipid metabolism, reducing lipid accumulation, and improving antioxidant and anti-inflammatory capacity [13,14,15,16]. However, the mechanism of the effect of MGF on cholesterol metabolism is less well studied at present and requires further research. Squalene epoxidase (sqle) is a key rate-limiting enzyme in cholesterol biosynthesis [17]. sqle catalyzes the first oxidative step in the formation of cholesterol, the oxidation of squalene to 2,3(S)-oxidosqualene. 2,3(S)-oxidosqualene is an important precursor for cholesterol synthesis [17]. 2,3(S)-oxidosqualene undergoes a series of enzymatic reactions to form cholesterol [17]. Previous studies have demonstrated that the overexpression of sqle results in increased levels of 2,3(S)-oxidosqualene and an increased cholesterol content, resulting in the development of hepatic damage, steatosis, and fatty liver formation [18]. The inhibition of sqle expression increases the squalene content, lowers the cholesterol content, and reduces hepatic lipid deposition [19]. Consequently, targeting sqle has emerged as an effective and appealing approach for cholesterol-lowering [20]. An increasing number of studies have shown that plant extracts have exhibited efficacy in inhibiting sqle activity and reducing cholesterol accumulation [21,22,23,24]. However, the relationship between MGF and sqle is unclear. Therefore, the hypothesis of the present study is that MGF may regulate cholesterol metabolism and thereby mitigate hepatic damage and abnormal lipid accumulation in fish.

Channel catfish (*Ictalurus punctatus*) is a world-wide aquaculture species, and in China, it had an annual production of 0.4 million tons in 2022 [25]. In the culture of aquaculture, high-starch diets lead to abnormally high cholesterol levels in channel catfish, they and will face an excessive accumulation of liver lipids and liver damage [26]. In our study, we investigated the impact of MGF on cholesterol metabolism-related genes of channel catfish, including synthesis-related genes (*hmgcr* and *sqle*), transcription factors of cholesterol metabolism-related genes (*srebp2* and *sp1*), transport-related genes (*ldlr* and *abca1*), and catabolism-related genes (*cyp7a1*), to elucidate the mechanisms underlying the MGF-mediated regulation of cholesterol. Furthermore, we found that MGF inhibited the gene expression level of *sqle* to the largest degree. In fish, Mouse hepatocytes (NCTC 1469) cells were also used to study liver metabolism; for example, in Gong et al. and Yan et al.’s studies, NCTC 1469 cells were used to study glucose metabolism and lipid metabolism in zebrafish liver [27,28]. We further verified the effect of MGF on *sqle* gene expression and cholesterol content in primary hepatocytes and NCTC 1469 cells. Interactions between small molecules and biomolecular targets can be predicted through molecular docking techniques. We investigated the possible molecular role between MGF and sqle by the molecular docking technique. Therefore, the present study set out to explore possible mechanisms to determine that MGF improves cholesterol metabolism and protects liver health in fish. These findings could offer valuable insights for modulating cholesterol metabolism in fish.

## 2. Materials and Methods

### 2.1. Experimental Diets

The present study formulated four isonitrogenous and isolipidic diets: a control diet (NCD), a high-starch diet (HCD), an HCD supplemented with 100 mg/kg MGF (100 MGF), and an HCD supplemented with 500 mg/kg MGF (500 MGF). The main protein sources included Peruvian fish meal, cottonseed protein concentrate, and soybean meal, and the main lipid sources were fish oil and soybean oil. The formulation and nutrition composition of the experimental diets are presented in Table 1. The various raw materials were precisely weighed based on the formulation, pulverized, and sieved through a 60-mesh screen. Subsequently, they were thoroughly mixed with water before being pelletized using a screw press pelletizer (SLR-150, Fishery Machinery and Instrument Research Institute, Shanghai, China).

### 2.2. Fish and Feeding Trial

Channel catfish were obtained from Zhengda Aquatic Products Co., Ltd. (Huanggang, China). Prior to the experiment, the fish were fed the control diet and acclimated to the experimental conditions for two weeks in a recirculating water system tank with a volume of 327 L. The similar-sized healthy fish (a total of 240 fishes) with an average weight of 35.5 ± 0.1 g were randomly allocated to 12 tanks at a stocking density of 20 fish per tank. Each group was randomly assigned three tanks, and the fish were fed twice daily for eight weeks. The water temperature (30.0 ± 1.5 °C), dissolved oxygen levels (7.1 ± 0.5 mg/L), pH values (7.1 ± 0.15), and ammonia nitrogen concentrations (0.17 ± 0.03 mg/L) were diligently monitored and consistently maintained on a daily basis throughout the entire feeding period.

### 2.3. Sample Collection

At the end of the feeding trial, the channel catfish were subjected to a 6 h fasting period and subsequently weighed in bulk. Prior to sampling, seven fish were randomly selected from each tank and euthanized using MS-222 (60 mg/L, Sigma, St. Louis, MO, USA). Two fish were frozen at −80 °C for body composition analysis (*n* = 6 per group). Two fish were used for biometric measurements (*n* = 6 per group), and the fish were individually measured for liver weight, body weight, and length. Two fish were used for the analysis of blood and liver indicators (*n* = 6 per group), 0.2% heparin was used as an anticoagulant, blood was collected from the caudal vein with 2 mL syringes and then centrifuged at 3000× *g* (4 °C) for 10 min, the supernatant was collected as plasma, and then it was stored at −80 °C. After blood sampling, the fish were dissected and the liver was collected. One part of the liver was fixed in a 4% paraformaldehyde solution, while the other part was stored at −80 °C until further use.

The growth-related parameters were calculated as follows:

WGR (%) = (final body weight (g) − initial body weight (g))/initial body weight (g) × 100;

SGR (%/d) = [Ln (final body weight) − Ln (initial body weight)]/days × 100;

HSI (%) = liver weight/whole body weight × 100;

CF (g/cm^3^) = whole body weight/(body length)^3^ × 100

FR (%BW/d) = total dry feed intake/[days × (final body weight (g) + initial body weight (g))/2] × 100;

FCE (%) = (final body weight (g) − initial body weight (g))/total dry feed intake (g) × 100.

### 2.4. Analysis of the Chemical Composition of the Experimental Diets and Fish Whole Body

The proximate composition analysis of the diets and the whole body of catfish was conducted in duplicate and measured according to the standard procedures of AOAC (2006) [29]. Each diet group was crushed and blended well for subsequent use. Each group of two fish was weighed and placed in the same aluminum box, dried at 75 °C, and then crushed and blended well for subsequent use. The moisture content was determined by subjecting the samples to thermal drying in an oven at 105 °C until a constant weight was achieved. Crude protein was determined by Kjeldahl analyses (2300Kjeltec analyzer unit, Foss Tecator, Hilleroed, Sweden). The crude lipid content was determined through diethyl ether extraction utilizing a Soxtec system (Soxtec system HT6, Tecator, Haganas, Sweden). The ash content was measured by subjecting the samples to high temperatures (550 °C) for 16 h in a muffle furnace (KSY12D16, Yingshan, China). The determination of crude fiber in diets was carried out with a filter bag fiber analyzer (CXC-06, Jingfei, Hangzhou, China).

### 2.5. H&E Staining and Oil Red O Staining

The liver tissues were fixed in 4% paraformaldehyde for 24 h, dehydrated through gradient alcohol dehydration, cleared with xylene, embedded in paraffin, and sliced to a thickness of 4 μm using a Leica RM 2135 slicing machine (Leica Company, Wetzlar, Germany). The experimental methodology was as previously described; the liver samples were subjected to morphological and histopathological analysis using hematoxylin-eosin staining [3], as well as hepatic lipid analysis using oil red O staining [30]. The images were acquired using an automated digital slide scanner (Aperio VERSA 8, Wetzlar, Germany).

### 2.6. Primary Hepatocyte Isolation and Culture

Primary channel catfish hepatocytes were established according to a previously described method [31]. Briefly, the liver tissues of the fish were aseptically excised following a 12 h fasting period and subsequently cut into small fragments in phosphate buffer saline (PBS) (C3580-0500, VivaCell, Shanghai, China). The fragments were then transferred to Hank’s balanced salt solution (HBSS) (14185052, Gibco, Thermo Fisher Scientific, Waltham, MA, USA) supplemented with collagenase type IV (C8160, Solarbio, Beijing, China) for digestion at 28 °C for 40 min. The hepatocytes were passed through a 40 μm nylon filter and subsequently purified by centrifugation to isolate the hepatocytes. The isolated hepatocytes were resuspended in DMEM/F12 medium (12400024, Gibco, Thermo Fisher Scientific, Waltham, MA, USA). Finally, the hepatocytes were evenly distributed onto culture plates and incubated at 28 °C in a humidified incubator with 5% CO_2_ for 12 h. During this period, the pH of the medium was maintained between 7.3 and 7.6. NB-598 in an inhibitor of *sqle* with cholesterol-lowering effects [32]. The cultured cells were divided into four groups, namely, an NCM group with the addition of DMEM/F12 medium, an HCM group with the addition of DMEM/F12 medium with 20 mM glucose (G7021, Sigma, St. Louis, MO, USA), an HCM+MGF group with the addition of 1.25 μM MGF (SM8090, Solarbio, Beijing, China) to the HCM, and an HCM+NB-598 group with the addition of 10 μM NB-598 (HY-16343, Med Chem Express, New Zeeland, AU, USA) to the HCM. The hepatocytes were harvested for gene expression and TC content analyses after a 12 h incubation period.

### 2.7. Cell Line Source and Culture Conditions

Mouse hepatocytes (NCTC 1469) cells were purchased from Procell Life Science & Technology (CL-0407, Procell, Wuhan, China). The NCTC 1469 cells were cultured as previously described. The cells were seeded in DMEM medium (C3103-0500, VivaCell, Shanghai, China) supplemented with 10% horse serum (C2510-0500, VivaCell, Shanghai, China) in a humidified atmosphere of 5% CO_2_ at 37 °C. The cells were seeded at an appropriate density in accordance with the specific experimental design. The cultured cells were divided into five groups, namely, an NCM group with the addition of M-199 medium (C3031-0500, VivaCell, Shanghai, China), an HCM group with the addition of M-199 medium with 20 mM glucose, an HCM+MGF group with the addition of 1.25 μM MGF to the HCM, an HCM+NB-598 group with the addition of 10 μM NB-598 to the HCM, and an HCM+MGF+NB-598 group with the addition of 1.25 μM MGF and 10 μM NB-598 to the HCM. The hepatocytes were harvested for gene expression and TC content analyses after a 12 h incubation period.

### 2.8. Cell Viability Assay

The cell viability was evaluated using the Cell Counting Kit-8 (CCK-8) (CA1210, Solarbio, Beijing, China) assay in accordance with the manufacturer’s protocol [33,34]. Briefly, NCTC 1469 cells were seeded in a 96-well plate at a density of 5000 cells/mL and subsequently treated with varying concentrations of MGF (0 μM, 1.25 μM, 2.5 μM, 5 μM, 10 μM, and 20 μM) for a duration of 12 h. Following the incubation period, each well was supplemented with 10 μL CCK-8 solution, and the cells were incubated under dark conditions at 37 °C for 1 h. The absorbance at 450 nm was subsequently measured using a microplate reader (PowerWave XS, BioTek, Winooski, VT, USA).

### 2.9. Plasma, Liver, and Cell Biochemical Analyses

Plasma glucose levels (GLU) were measured using a glucose kit (F006-1-1). Plasma alanine aminotransferase activity (ALT) was measured using an alanine aminotransferase assay kit (C009-2-1). Plasma aspartate aminotransferase activity (AST) was determined using an aspartate aminotransferase assay kit (C010-2-1). Plasma high-density lipoprotein cholesterol levels (HDL) were measured using a high-density lipoprotein cholesterol assay kit (A112-1-1). Plasma low-density lipoprotein cholesterol levels (LDL) were measured using a low-density lipoprotein cholesterol assay kit (A113-1-1). Plasma total bile acids (TBA) levels were measured using the total bile acids assay kit (E003-2-1). Plasma, tissue, and cell total cholesterol (TC) levels were measured using a total cholesterol assay kit (A111-1-1). Plasma and tissue triglyceride (TG) levels were measured using the triglyceride assay kit (A110-1-1). Tissue total antioxidant capacity (T-AOC) levels were measured using the total antioxidant capacity assay kit (A015-2-1). The above experimental kits were used at Nanjing Jiancheng Bioengineering Institute (Nanjing, China), and the experimental methodology was as previously described [35,36,37]. Tissue reactive oxygen species (ROS) levels were measured by the reactive oxygen species (KT86537, mskbio, Wuhan, China). Tissue and cell protein concentrations were determined using the Bradford method (P0006, Beyotime, Shanghai, China) [38,39,40]. All measurements adhered to the manufacturer’s instructions.

### 2.10. Quantitative Real-Time PCR (qPCR)

Total RNA was extracted from liver tissues using the Trizol reagent (Ambion Life Technologies, Carlsbad, CA, USA), followed by reverse transcription into cDNA using the M-MLV First-Stand Synthesis Kit (Invitrogen, Shanghai, China). The samples were prepared in duplicate and subjected to RT-qPCR analysis with SYBR Green I Master Mix (Roche Diagnostics, Indianapolis, IN, USA) on a LightCycle 480 II system (Roche, Basel, Switzerland). β-actin and ef1α served as the internal reference gene. Finally, the results were analyzed using the 2^−ΔΔCt^ method for the calculation of relative expression levels. Primer sequences are provided in Table 2 and Table 3.

### 2.11. Immunofluorescence Assay

Immunofluorescence assays were conducted following a previously established protocol [41]. The primary antibody was anti-*sqle* (12544-1-AP, Proteintech, Rabbit, Hubei, China). Subsequently, visualization was achieved by employing an Alexa Fluor 568-conjugated secondary antibody (A11036, Thermo Fisher Scientific, rabbit, Waltham, MA, USA) specific to immunoglobulins. Nuclei were stained with Hoechst 33342 (H21492, Thermo Fisher Scientific, Waltham, MA, USA). The images were acquired using a Leica laser-scanning confocal microscope (SP8 DLS, GER), processed with Imaris Viewer software 9.5.1 (Oxford Instruments, Oxford, UK). The image color channels were split using ImageJ (National Institutes of Health, Bethesda, MD, USA), the red channel 8-bit black and white image was selected, the average grey value of each image was measure, which also represents the average fluorescence intensity of the red fluorescence on that image, and finally, the relative fluorescence intensity of each group of images was calculated using the normalization method.

### 2.12. Molecular Docking

The MGF (ID: 5281647) data were retrieved from the PubChem database (https://pubchem.ncbi.nlm.nih.gov, accessed on 20 January 2024). The homology modeling was conducted due to the unavailability of the crystal structure of the channel catfish sqle in the Protein Data Bank (PDB). Utilizing the NCBI BLAST (https://blast.ncbi.nlm.nih.gov/Blast.cgi, accessed on 20 January 2024) facilitated the identification of the model exhibiting the highest sequence homology to channel catfish sqle in PDB. Human sqle (ID: 6C6N) exhibits the highest degree of homology, with a similarity of up to 72.81% compared to the channel catfish sqle. The sqle amino acid sequence (XP_017308548.1) retrieved from Genbank (https://www.ncbi.nlm.nih.gov/genbank/, accessed on 3 February 2024) was employed for homology modeling using SwissModel (https://swissmodel.expasy.org/, accessed on 7 February 2024). Subsequently, the most optimal conformation obtained from the homology modeling results was selected as the receptor protein for subsequent molecular docking. Finally, the model was validated using the PROCHECK and ERRAT programs (https://saves.mbi.ucla.edu/, accessed on 7 February 2024). The ligand (MGF) and sqle protein underwent energy minimization, dehydration, and hydrogenation using AutoDock 4.2.6, with 500 dockings performed. The conformation with the lowest docking energy was selected as the most probable binding state, followed by visualization using PyMOL 2.4.

### 2.13. Statistics

The experimental data were analyzed using SPSS 27.0 (IBM, Chicago, IL, USA) statistical software and subjected to one-way analysis of variance (ANOVA). Prior to the analysis, the normality of the data was assessed using the Shapiro–Wilk test, and the homogeneity of variance was evaluated using Levene’s test for the equality of variances. A probability value of *p* < 0.05 was considered statistically significant. GraphPad Prism 9 software (GraphPad Software, Boston, MA, USA) was employed for generating the graphical representation of this experiment.

## 3. Results

### 3.1. MGF Improves the Growth Performance of Channel Catfish

**Figure 1:**
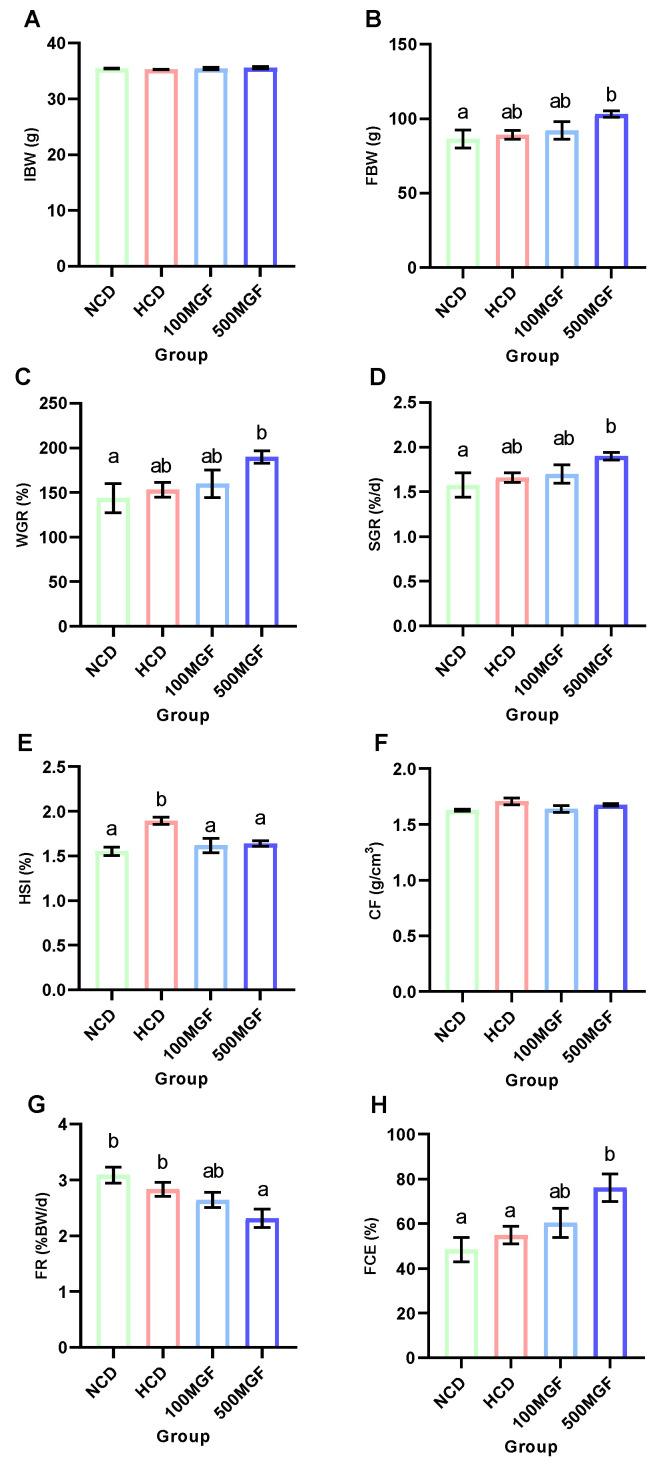
Effects of dietary MGF supplementation on the growth performance and feed utilization of channel catfish. (**A**) Initial body weight (IBW); (**B**) Final body weight (FBW); (**C**) Weight gain rate (WGR); (**D**) Specific growth rate (SGR); (**E**) Hepatosomatic index (HSI); (**F**) Condition factor (CF); (**G**) Feeding rate (FR); (**H**) Feed conversion efficiency (FCE). Values are expressed as means ± SEMs (IBW, FBW, WGR, SGR, FR, and FCE, *n* = three replicates; HSI and CF, *n* = three replicates; two fish were pooled in each replicate). Bars assigned different superscripts are significantly different (*p* < 0.05). The colors of the different bars represent the different groups: NCD group (green), HCD group (red), 100 MGF group (blue), and 500 MGF group (dark blue).

The effects of dietary MGF supplementation on the growth performance and feed utilization of channel catfish are shown in Figure 1. At the end of the feeding trial, the final body weight (FBW) (*p* = 0.031), weight gain rate (WGR) (*p* = 0.031), and specific growth rate (SGR) (*p* = 0.039) of the 500 MGF group were significantly higher than those of the NCD group (Figure 1B–D). The hepatosomatic index (HSI) of the NCD, 100 MGF, and 500 MGF groups exhibited a significantly lower value compared to that of the HCD group (*p* = 0.009) (Figure 1E). However, there was no significant difference in the condition factor (CF) among the four groups (*p* = 0.142) (Figure 1F). The feeding rate (FR) of the 500 MGF group was significantly lower than that of the NCD and HCD groups (*p* = 0.028) (Figure 1G), but the feed conversion efficiency (FCE) of the 500 MGF group was significantly higher than that of the NCD and HCD groups (*p* = 0.040) (Figure 1H).

### 3.2. Proximate Body Composition

**Figure 2:**
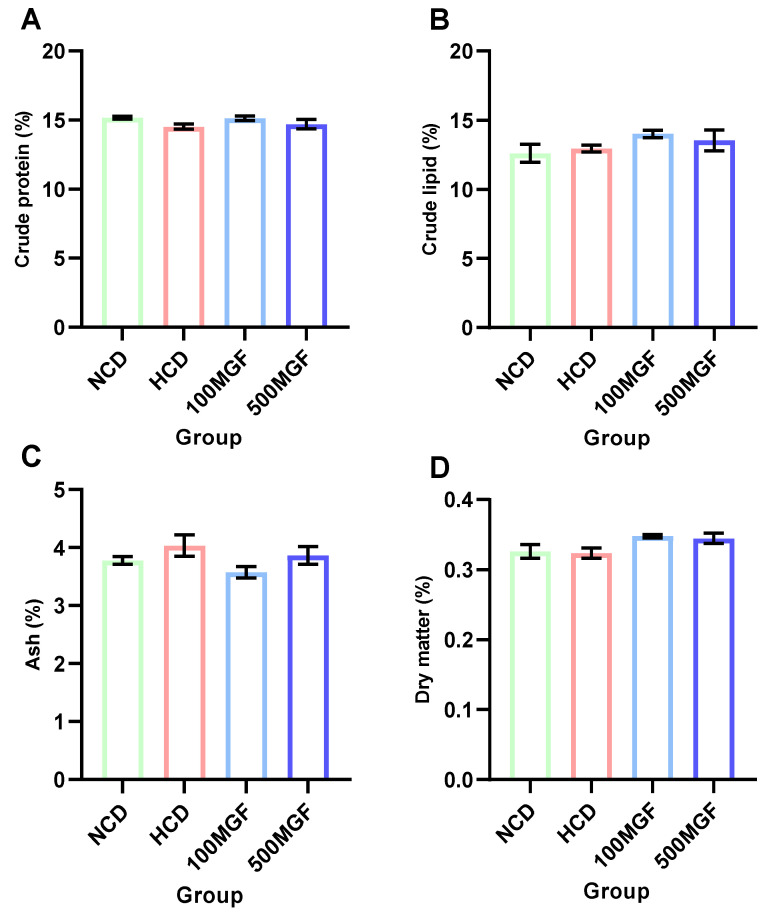
The proximate body composition of channel catfish. (**A**) Crude protein content; (**B**) Crude lipid content; (**C**) Ash content; (**D**) Dry matter content. Values are expressed as the means ± SEMs (*n* = three replicates; two fish were pooled in each replicate). The colors of the different bars represent the different groups: NCD group (green), HCD group (red), 100 MGF group (blue), and 500 MGF group (dark blue).

The dietary addition of MGF had no effect on the proximate composition analysis (Figure 2). According to the results of the whole-body proximate composition analysis, there were no significant differences in the crude lipid (*p* = 0.224), crude protein (*p* = 0.221), crude ash, (*p* = 0.181) and moisture contents (*p* = 0.095) between the NCD, HCD, 100 MGF, and 500 MGF groups.

### 3.3. MGF Effects on HCD-Induced Hepatic Damage

**Figure 3:**
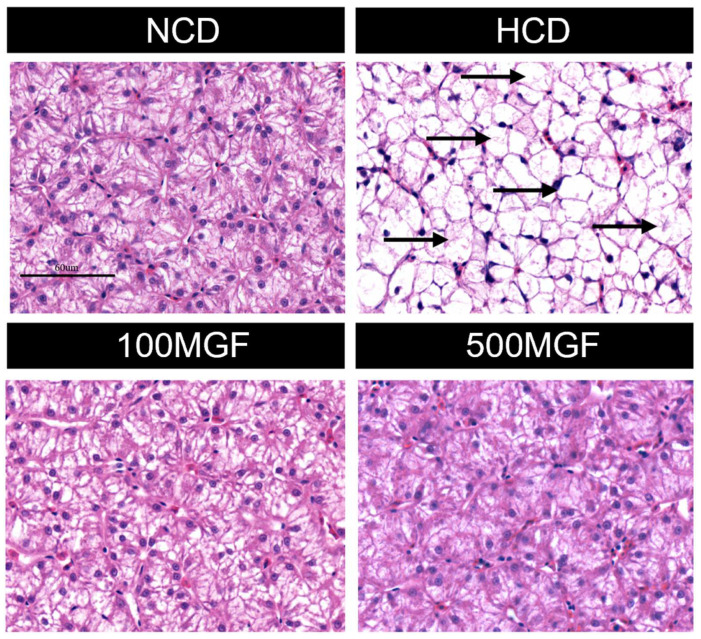
Effects of dietary MGF supplementation on the histological structure of the liver (H&E stain) in channel catfish (scale bar = 60 µm). The black arrow indicates a significant loss of intracellular nuclei.

**Figure 4:**
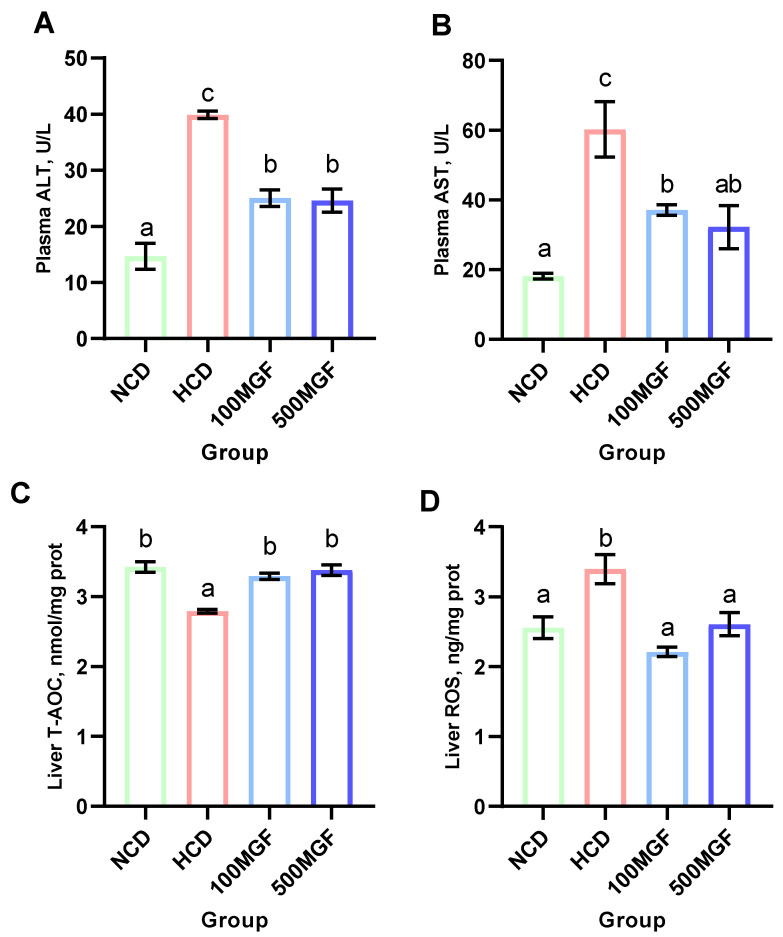
Effects of dietary MGF supplementation on the liver health of the channel catfish. (**A**) Plasma alanine aminotransferase activity (ALT); (**B**) Plasma aspartate aminotransferase activity (AST); (**C**) Liver total antioxidant capacity (T-AOC); (**D**) Liver reactive oxygen species (ROS). Values are expressed as means ± SEMs (ALT, AST, T-AOC, and ROS, *n* = three replicates; two fish were pooled in each replicate). Bars assigned different superscripts are significantly different (*p* < 0.05). The colors of the different bars represent the different groups: NCD group (green), HCD group (red), 100 MGF group (blue), and 500 MGF group (dark blue).

According to the histological analysis of the liver sections stained with H&E, it was observed that the HCD group exhibited more pronounced hepatocyte vacuolization characterized by a significant loss of intracellular nuclei and an increased severity of hepatocyte enlargement compared to the NCD, 100 MGF, and 500 MGF groups (Figure 3). The hepatic total antioxidant capacity (T-AOC) was significantly lower in the HCD group compared to that in the NCD, 100 MGF, and 500 MGF groups (*p* = 0.000) (Figure 4C). Additionally, the plasma alanine aminotransferase (ALT) activity (*p* = 0.000), plasma aspartate aminotransferase (AST) activity (*p* = 0.003), and liver reactive oxygen species (ROS) levels (*p* = 0.004) were significantly higher in the HCD group than in the NCD, 100 MGF, and 500 MGF groups (Figure 4A,B,D). Consequently, in comparison to NCD diets, HCD diets elicit hepatic damage in channel catfish, which can be alleviated through the supplementation of MGF.

### 3.4. MGF Reduces the High Blood Glucose and Lipid Levels Caused by an HCD Diet

**Figure 5:**
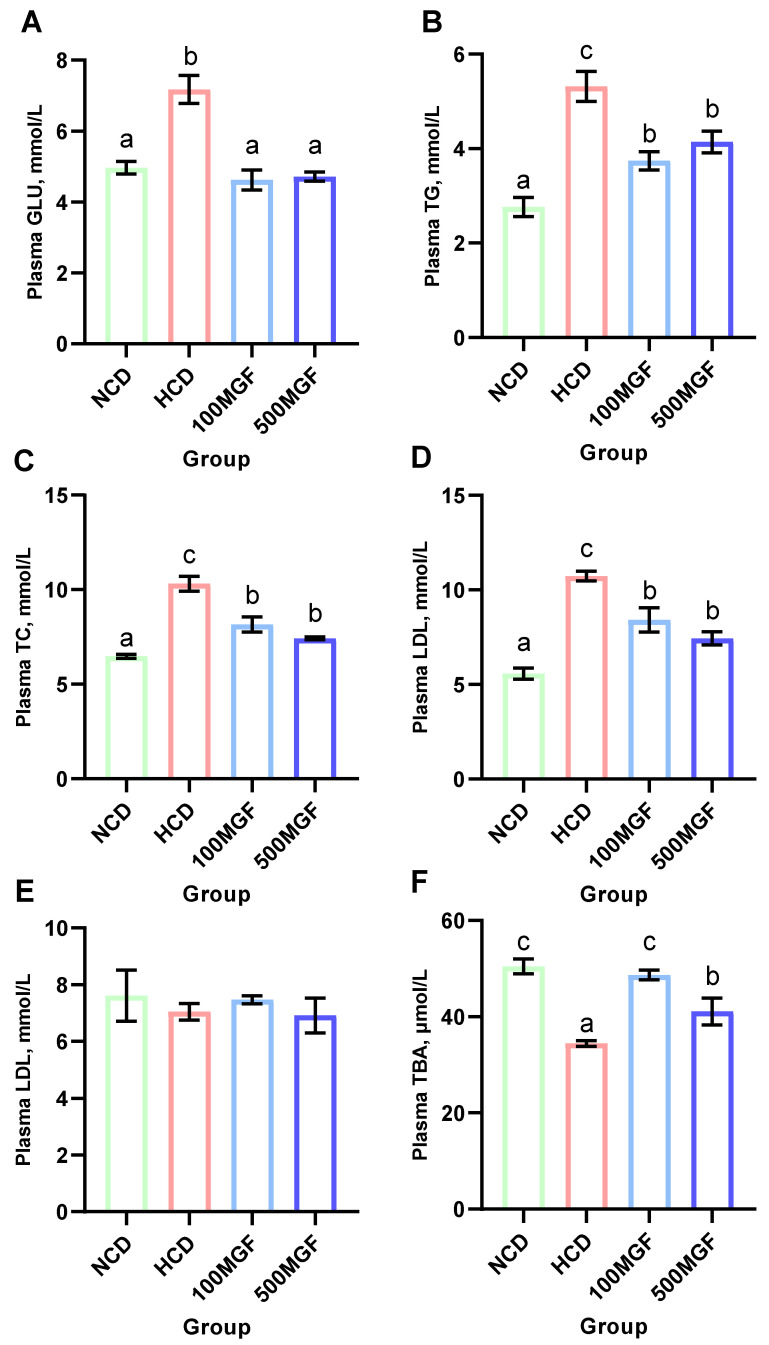
Effects of dietary MGF supplementation on the plasma glucose and lipid levels of the channel catfish. (**A**) Plasma glucose (GLU); (**B**) Plasma triglyceride (TG); (**C**) Plasma total cholesterol (TC); (**D**) Plasma low-density lipoprotein cholesterol (LDL); (**E**) Plasma high-density lipoprotein cholesterol (HDL); (**F**) Plasma total bile acid (TBA). Values are expressed as means ± SEMs (*n* = three replicates; two fish were pooled in each replicate). Bars assigned different superscripts are significantly different (*p* < 0.05). The colors of the different bars represent the different groups: NCD group (green), HCD group (red), 100 MGF group (blue), and 500 MGF group (dark blue).

The results indicated that the plasma glucose (GLU) levels were significantly lower in the NCD, 100 MGF, and 500 MGF groups compared to those in the HCD group (*p* = 0.000) (Figure 5A). Additionally, the plasma triglyceride (TG) (*p* = 0.001), total cholesterol (TC) (*p* = 0.000), and low-density lipoprotein cholesterol (LDL) (*p* = 0.000) levels were significantly lower in the NCD, 100 MGF, and 500 MGF groups compared to those in the HCD group (Figure 5B–D). Plasma high-density lipoprotein cholesterol (HDL) was not significantly changed (*p* = 0.797) (Figure 5E). Plasma total bile acid (TBA) was significantly lower in the HCD group compared to that in the NCD group, and the 100 MGF and 500 MGF groups were not significantly different compared to the NCD group (*p* = 0.001) (Figure 5F). Consequently, in comparison to NCD diets, HCD diets induced aberrant blood glucose and lipid levels in channel catfish, which can be mitigated by the supplementation of MGF.

### 3.5. MGF Alleviates HCD-Induced Hepatic Lipid Accumulation

**Figure 6:**
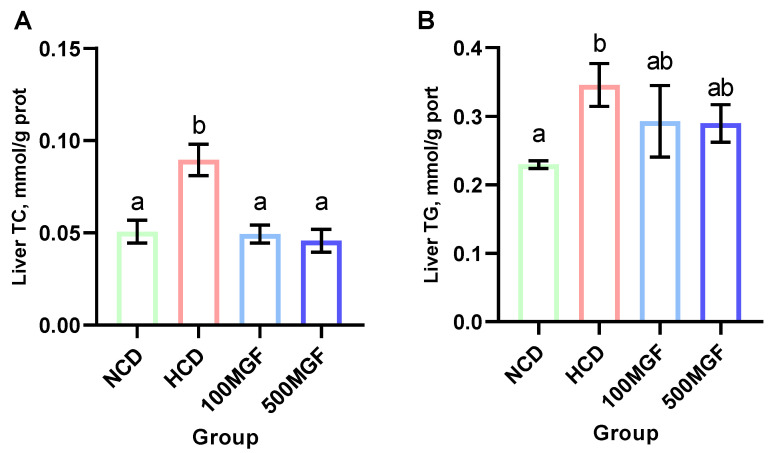
Effects of dietary MGF supplementation on the hepatic lipid content of the channel catfish. (**A**) Liver total cholesterol (TC); (**B**) Liver triglyceride (TG). Values are expressed as means ± SEMs (TC and TG, *n* = three replicates; two fish were pooled in each replicate). Bars assigned different superscripts are significantly different (*p* < 0.05). The colors of the different bars represent the different groups: NCD group (green), HCD group (red), 100 MGF group (blue), and 500 MGF group (dark blue).

**Figure 7:**
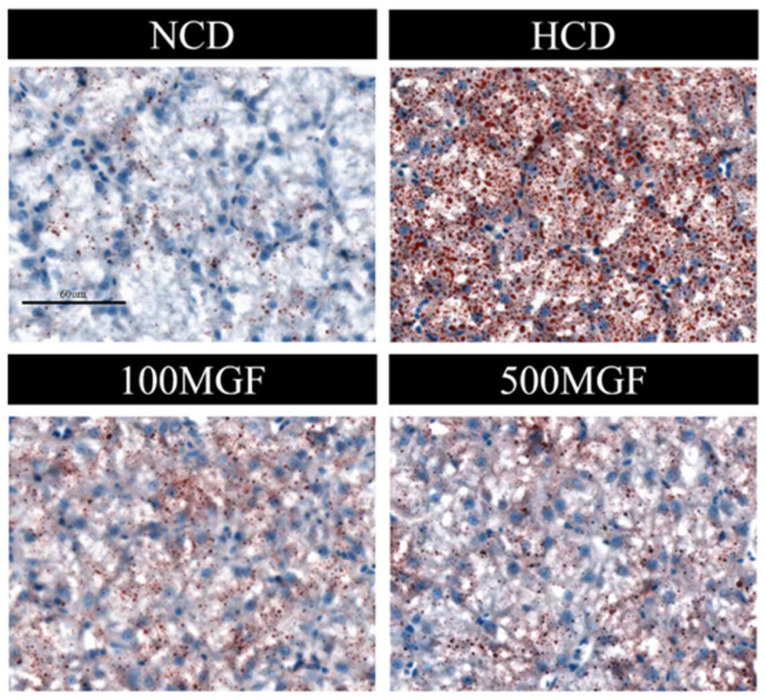
Effects of dietary MGF supplementation on liver lipid droplets (oil red O staining) in channel catfish (scale bar = 60 µm).

The results showed that the liver TC and TG levels were significantly increased in the HCD group compared with those of the NCD group (Figure 6A,B). The supplementation of MGF led to a noteworthy reduction in the liver TC content (*p* = 0.005) (Figure 6A), while no significant difference was observed in the TG content (*p* = 0.190) (Figure 6B). Furthermore, we conducted histological analysis using hepatic oil red O staining sections to evaluate fat accumulation (Figure 7). Our findings revealed a significant increase in fat droplet deposition in the HCD group compared to the NCD group, while supplementation with MGF effectively mitigated high-starch-diet-induced fat droplet accumulation. Overall, these findings suggest that MGF exerts a significant impact on hepatic TC levels by effectively reducing them and concurrently mitigating lipid accumulation in channel catfish.

### 3.6. MGF Regulates Hepatic Cholesterol Metabolism

**Figure 8:**
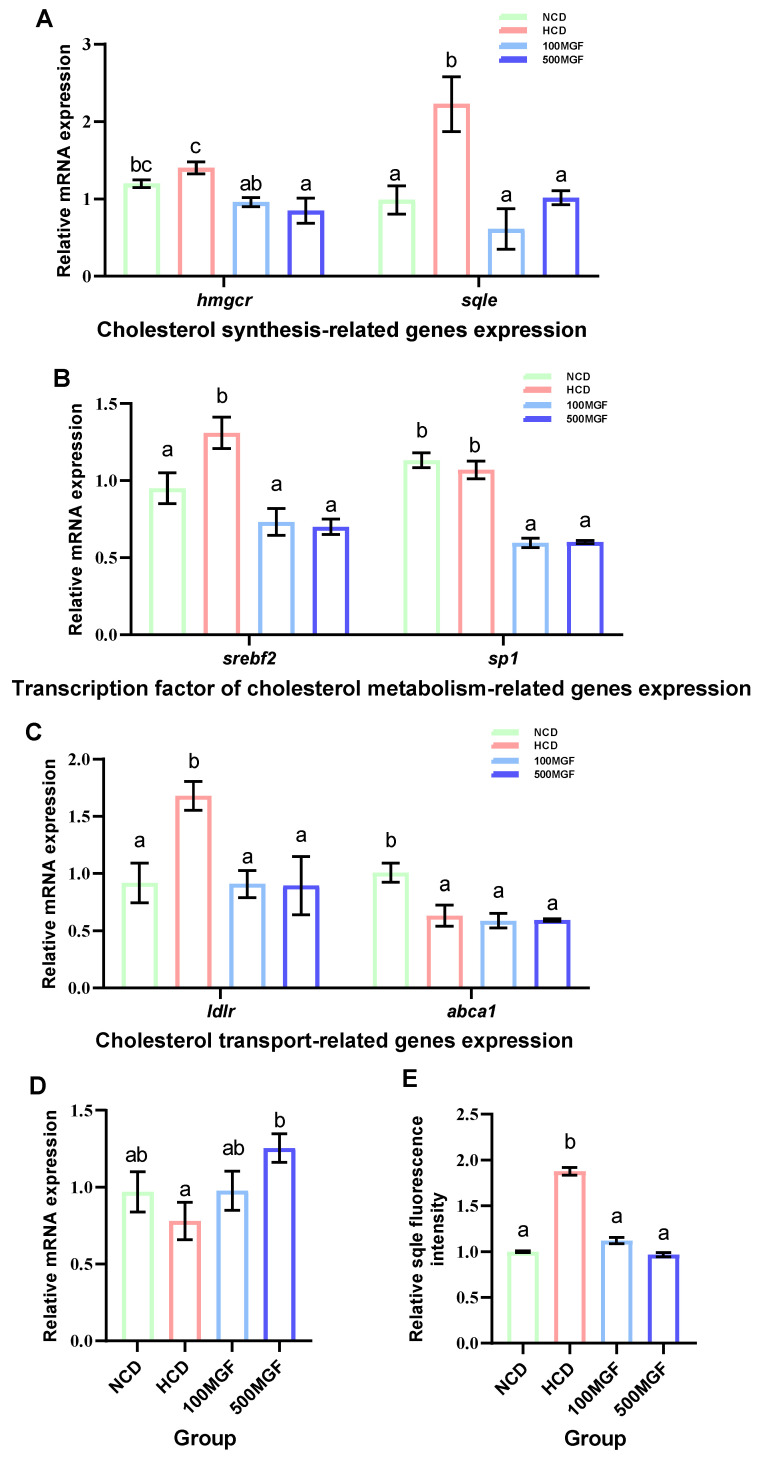
Effects of dietary MGF supplementation on the expression levels of cholesterol metabolism-related genes and protein expression in the liver of channel catfish. (**A**) Cholesterol synthesis-related genes expression; (**B**) Transcription factor of cholesterol metabolism-related genes expression; (**C**) Cholesterol transport-related genes expression; (**D**) Cholesterol catabolism-related genes expression; (**E**) Quantification of the *sqle* relative fluorescence intensity. Values are expressed as means ± SEMs (Cholesterol synthesis-related genes, transcription factor of cholesterol metabolism-related genes, cholesterol transport-related genes and cholesterol catabolism-related genes expression; *n* = three replicates; two fish were pooled in each replicate; *sqle* immunofluorescence staining/quantification, *n* = three replicates). Bars assigned different superscripts are significantly different (*p* < 0.05). The colors of the different bars represent the different groups: NCD group (green), HCD group (red), 100 MGF group (blue), and 500 MGF group (dark blue).

**Figure 9:**
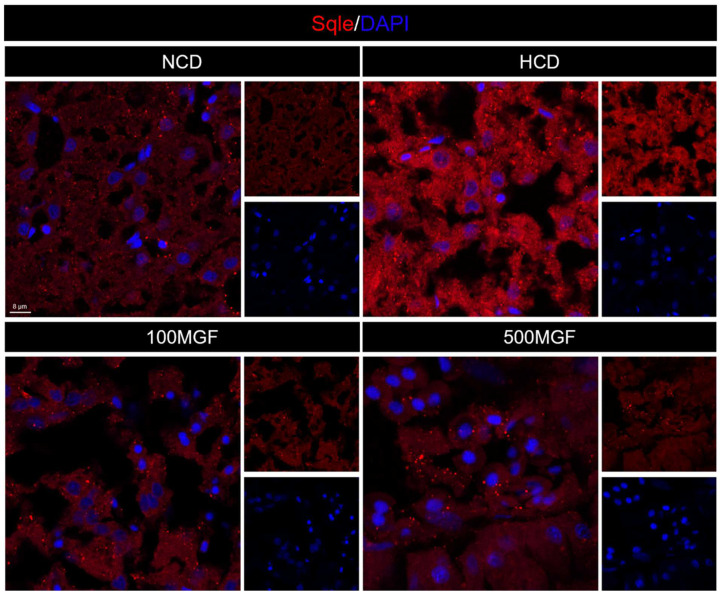
Effects of dietary MGF supplementation on the expression levels of Sqle protein expression in the liver of channel catfish. Hepatic Sqle (Red) staining images (scale bar = 8 µm).

Effects of dietary MGF supplementation on the expression levels of cholesterol metabolism-related genes and protein expression in the liver of channel catfish, as shown in Figure 8. The present results showed that the expression level of *hmgcr* did not show any significant difference between the NCD and HCD groups, while the 100 MGF or 500 MGF groups had a significant decrease of 1.46-fold or 1.65-fold in the *hmgcr* expression level compared to that of the HCD group (*p* = 0.017) (Figure 8A). The expression level of sqle was significantly increased in the HCD group compared to that of the NCD group, while the 100 MGF or 500 MGF groups had a significantly decreased (by 3.64-fold or 2.20-fold) *sqle* expression level compared to that of the HCD group (*p* = 0.008) (Figure 8A). The Sqle protein expression level was significantly increased in the HCD group compared to that in the NCD group, while the 100 MGF or 500 MGF groups had significantly decreased (by 1.67-fold or 1.94-fold) sqle protein levels compared to that of the HCD group (*p* = 0.000) (Figure 8E and Figure 9). *srebf2* and *sp1* are important transcription factors in cholesterol metabolism. The expression level of *srebf2* was significantly increased in the HCD group compared with that in the NCD group (Figure 8B). When compared to that in the HCD group, the expression of *srebf2* (*p* = 0.004) and *sp1* (*p* = 0.000) was significantly decreased in the 100 MGF and 500 MGF groups (Figure 8B). The genes *ldlr* and *abca1* are implicated in the regulation of cholesterol transport. The expression level of *ldlr* was significantly decreased in the NCD, 100 MGF, and 500 MGF groups compared to that in the HCD group (*p* = 0.034) (Figure 8C). The expression level of *abca1* was significantly decreased in the HCD, 100 MGF, and 500 MGF groups compared to that in the NCD group (*p* = 0.008) (Figure 8C). Cholesterol catabolism is regulated by the key rate-limiting enzyme *cyp7a1*. Our results demonstrated a significantly increased expression level of *cyp7a1* in the 500 MGF group compared to that in the HCD group (*p* = 0.042) (Figure 8D).

**Figure 10:**
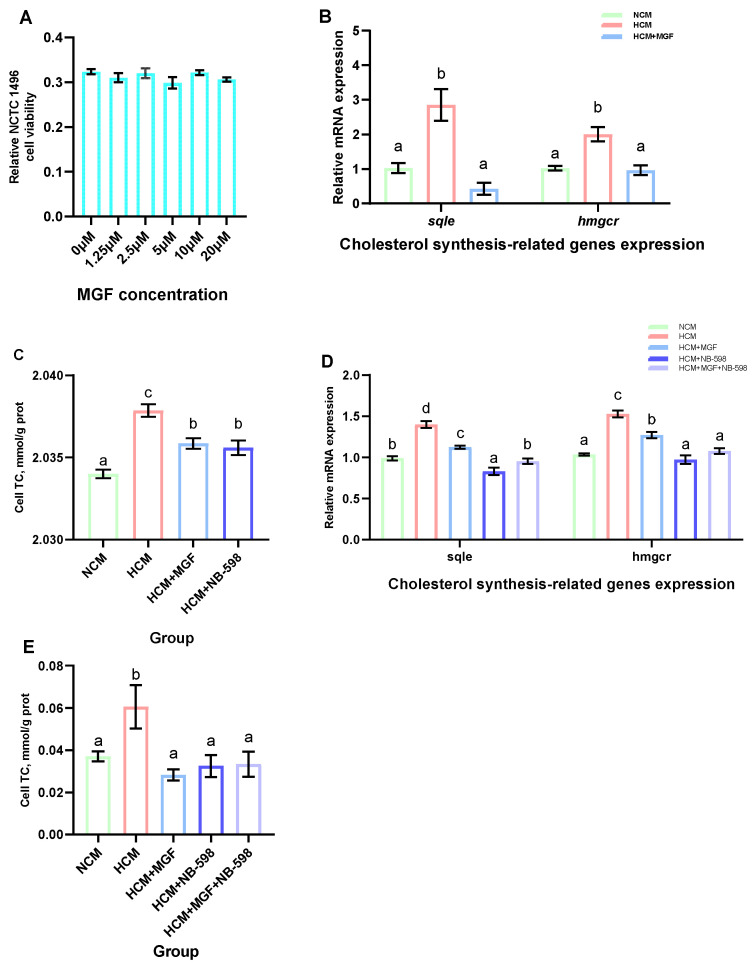
Effects of adding MGF on primary hepatocytes of channel catfish and NCTC 1469 cells. (**A**) NCTC 1469 cells viability; (**B**) Expression levels of cholesterol synthesis-related genes in the primary hepatocytes of channel catfish; (**C**) Total cholesterol (TC) content in the primary hepatocytes of channel catfish; (**D**) Expression levels of cholesterol synthesis-related genes in NCTC 1469 cells; (**E**) Total cholesterol (TC) content in NCTC 1469 cells. Values are expressed as means ± SEMs (NCTC 1469 cells viability, *n* = six replicates; Expression levels of cholesterol synthesis-related genes in the primary hepatocytes of channel catfish, *n* = three replicates; Expression levels of cholesterol synthesis-related genes in NCTC 1469 cells, *n* = four replicates; TC content in the primary hepatocytes of channel catfish and NCTC 1469 cells, *n* = three replicates). Bars assigned different superscripts are significantly different (*p* < 0.05). The colors of the different bars represent the different groups: primary hepatocytes of channel catfish are grouped in Figure 10B,C as the NCM group (green), HCM group (red), HCM+MGF group (blue), and HCM+NB-598 group (dark blue); NCTC 1469 cells are grouped in Figure 10D,E as the NCM group (green), HCM group (red), HCM+MGF group (blue), HCM+NB-598 group (dark blue), and HCM+MGF+NB-598 group (lavender).

To investigate the regulatory effects of MGF on cholesterol metabolism and its potential to reduce cholesterol accumulation, we further explored this in primary channel catfish hepatocytes and NCTC 1469. First, various concentrations of MGF were added to the NCTC 1469 cell culture medium and cultured for 12 h to assess cellular viability. The results showed that there was no significant difference in cell viability between the groups of NCTC 1469 cells added with 0 μM, 1.25 μM, 2.5 μM, 5 μM, 10 μM, and 20 μM MGF (*p* = 0.265) (Figure 10A). Next, we validated this in the primary hepatocytes of channel catfish, and the results showed that the expression levels of *sqle* and *hmgcr* were significantly increased by the stimulation of the high-carbohydrate medium; the addition of the MGF group to a high-carbohydrate medium resulted in a 5.04-fold and 2.08-fold decrease in *sqle* (*p* = 0.030) and *hmgcr* (*p* = 0.005) expression levels, respectively (Figure 10B). The cholesterol content of the primary hepatocytes increased significantly under the stimulation of a high-carbohydrate medium, and the addition of MGF reduced its content and achieved the same effect as the sqle inhibitor NB-598 (*p* = 0.000) (Figure 10C). Meanwhile, our validation in NCTC 1469 demonstrated that the expression levels of sqle and hmgcr were significantly increased under the stimulation of a high-carbohydrate medium. The expression of sqle (*p* = 0.000) and hmgcr (*p* = 0.000) was decreased by 1.28-fold and 1.19-fold, respectively, with the addition of the MGF group to a high-carbohydrate medium, but the extent of the reduction was less than that in the group with the addition of NB-598 (Figure 10D). The cholesterol content of the NCTC 1469 increased significantly under the stimulation of a high-carbohydrate medium, and the addition of MGF reduced its content and achieved the same effect as the sqle inhibitor NB-598 (*p* = 0.024) (Figure 10E).

### 3.7. Molecular Docking Result

**Figure 11:**
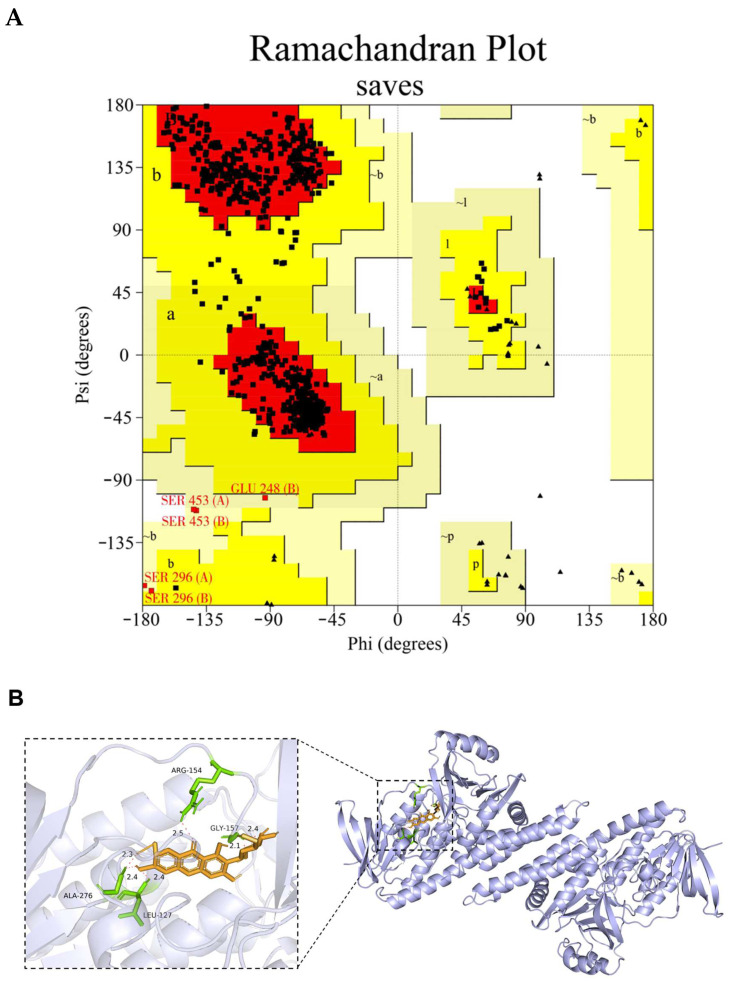
The homology model structure of the Sqle of channel catfish was subjected to reliability validation and molecular docking analysis. (**A**) The homology model’s Ramachandran plot (The red area is the optimal region where amino acid residues are located, the dark yellow area is the other permissible region where amino acid residues are located, and the light-yellow area is the maximum permissible region where amino acid residues are located. Of these, Amino acid residues (SER 453 (A/B), SER 296 (A/B) and GLU 248 (B)) are located within the maximum permitted region); (**B**) MGF and Sqle molecular docking analysis.

Before docking, the homology model of the channel catfish Sqle was established. The subsequent reliability validation was conducted, and the model evaluation revealed that 93.3% of the amino acid residues were located within the optimal region in the pull graph generated by PROCHECKserver (https://saves.mbi.ucla.edu/, accessed on 7 February 2024), 6.0% resided in other permissible regions, and only 0.6% and 0.0% were observed in the maximum allowable and disallowed regions, respectively (Figure 11A). Moreover, the ERRAT score was 96.45%, which means that the calculated error was below the 99% rejection limit for 96.45% of the residues. These findings strongly support the rationality of the constructed Sqle model for subsequent analysis in channel catfish. The interaction between MGF and Sqle in channel catfish was investigated through molecular docking analysis. The results revealed a strong binding affinity of MGF towards Sqle in channel catfish. The binding sites for MGF and Sqle mainly involve ARG-154, GLY-157, ALA-276, and LEU-127, with a binding energy of −4.62 kJ/mol (Figure 11B).

## 4. Discussion

Related studies have shown that the addition of botanical extracts to enhance starch utilization in fish can significantly improve their growth performance [42,43]. However, there is limited research on the effects of MGF on growth performance. Several studies have reported a significant reduction in the body weight of rats and mice in the diabetes model compared to the normal group [44,45]. Treatment with MGF resulted in a substantial increase in their body weight. Furthermore, MGF administration also demonstrated an ability to enhance the body weight in rats in the NASH model [46]. This finding is consistent with the present results of our study, as we observed a significant improvement in growth performance such as weight gain, the specific growth rate, and feed efficiency in the 500 MGF group compared to the NCD group for channel catfish.

The current results suggest that MGF could mitigate hepatic damage and ameliorate the lipid accumulation induced by a high-starch diet. First, we found that channel catfish showed symptoms of liver damage when dietary starch levels were increased, and this phenomenon was similarly seen in other fish species [6,47]. The initial mechanism leading to hepatocellular damage stems from the toxic effects of excess lipids [8]. Accumulating evidence suggests that the total number of triglycerides stored in hepatocytes is not the only determinant of lipotoxicity, but rather, specific lipid classes exert detrimental effects on hepatocytes, including cholesterol [9,48]. The abnormal accumulation of free cholesterol can damage hepatocytes by disrupting mitochondrial and endoplasmic reticulum membrane integrity, inducing mitochondrial oxidative damage and endoplasmic reticulum stress, and promoting the production of toxic oxysterols [48,49]. In the current study, the addition of MGF decreased plasma ALT and AST activity, reduced liver pathological changes and ROS content, and increased T-AOC. MGF has the function of alleviating liver damage, but its mechanism of action is not clear [22,50,51]. Additionally, a high-starch diet induces lipid accumulation in fish, leading to the development of a fatty liver [5,47]. Relevant studies have reported that the dysregulation of hepatic cholesterol metabolism, characterized by abnormally elevated cholesterol levels, can contribute to the development of hepatic steatosis [52]. MGF has significant efficacy in reducing the accumulation of cholesterol in the body [13,14]. We observed that MGF significantly mitigated the elevation of cholesterol content and lipid deposition in both channel catfish and cell experiments induced by high-carbohydrate stimulation. Thus, we speculated that MGF could alleviate liver damage and ameliorate lipid accumulation by regulating cholesterol homeostasis.

One of the major causes of cholesterol homeostasis disruption in fish is increased cholesterol synthesis [52]. HMG-CoA reductase (hmgcr) and sqle emerge as two key enzymes in the synthesis of cholesterol [17]. In the current study, the gene expression of *hmgcr* and *sqle* decreased significantly after MGF supplementation, with a higher-fold decrease in *sqle* gene expression. In addition, the expression level of *sqle* was found to be positively correlated with cholesterol levels in both channel catfish and cell experiments, which is consistent with previous studies [13,42]. sqle is like a switch regulating cholesterol synthesis, making it an important target for cholesterol-lowering [19]. We therefore speculated that MGF may reduce cholesterol synthesis by regulating the expression of *sqle*. More and more studies have demonstrated the cholesterol-lowering function of MGF, while the mechanism remains a mystery [53]. Notably, the present experiments revealed that MGF significantly inhibited the increase in *sqle* expression caused by a high carbohydrate medium and was comparable to the sqle inhibitor NB-598. In addition, the stimulation of the nuclear translocation of srebf2 and sp1 enhances sqle expression, thereby resulting in intracellular cholesterol accumulation [54]. In this experiment, the addition of the MGF group significantly reduced the expression of *srebf2* and *sp1* compared to the addition of the HCD group. It is supposed that MGF may regulate the expression of *sqle* by repressing its transcription factor and thereby regulating *sqle*.

The discovery of a potential direct correlation between MGF and Sqle is noteworthy in our study. Molecular docking helps us to understand the interaction between the drug and the target molecule [55]. The molecular docking techniques have successfully identified the interaction of MGF with myosin, tumor necrosis factor (TNF-α), and matrix metalloproteinase-9 (MMP-9), elucidating potential mechanisms of molecular interactions [56,57]. The results of this experiment demonstrate a robust binding interaction between MGF and Sqle through molecular docking simulations, indicating that MGF may exert its inhibitory effect on Sqle activity by directly interacting with it. Overall, MGF may inhibit cholesterol accumulation by suppressing sqle expression, but further validation and more reliable evidence are needed in future investigations.

## 5. Conclusions

**Figure 12:**
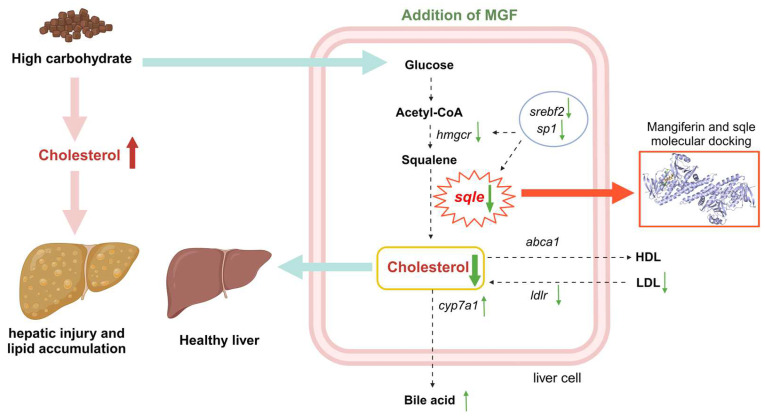
Working model depicting that the supplementation of mangiferin to a high-starch diet attenuates hepatic damage and lipid accumulation by modulating cholesterol metabolism. Green upward thin arrows indicate up-regulation of gene expression and green downward thin arrows indicate down-regulation of gene expression; red upward thick arrows indicate an increase in cholesterol content and green downward thick arrows indicate a decrease in cholesterol content.

MGF regulates *sqle* expression to maintain cholesterol homeostasis in vivo and attenuates liver damage and lipid accumulation by reducing cholesterol synthesis in the liver of channel catfish fed the high-starch diet (Figure 12). Furthermore, MGF facilitates the growth performance of channel catfish. These findings establish a robust foundation for the utilization of MGF as an advantageous reagent in safeguarding hepatic well-being and fostering growth in cultured fish.

## Figures and Tables

**Table 1:** Formulation and proximate composition of the experimental diets (% dry matter).

Ingredients	Experimental Diets			
	NCD	HCD	100 MGF	500 MGF
^1^ Fish meal	12.00	12.00	12.00	12.00
^2^ Cottonseed protein concentrate	28.00	28.00	28.00	28.00
^3^ Soybean meal	14.00	14.00	14.00	14.00
^4^ Soybean oil	2.20	2.20	2.20	2.20
^5^ Fish oil	2.20	2.20	2.20	2.20
^6^ Corn starch	18.00	36.00	36.00	36.00
^7^ Microcrystalline cellulose	18.00	0.00	0.00	0.00
^8^ Sodium carboxymethylcellulose	2.79	2.79	2.78	2.74
^9^ Monocalcium phosphate	1.50	1.50	1.50	1.50
^10^ Vitamin and mineral premix	1.20	1.20	1.20	1.20
^11^ Choline chloride	0.11	0.11	0.11	0.11
^12^ Mangiferin	0.00	0.00	0.01	0.05
**Proximate composition (% dry basis)**				
Crude protein	33.52	33.89	33.24	33.63
Crude lipid	4.97	4.81	4.79	5.09
Crude fiber	22.33	9.91	9.95	9.92
Moisture	10.96	7.74	6.15	7.02
Ash	6.55	6.64	7.00	6.88
^13^ Nitrogen free extract	21.67	37.01	38.87	37.46

Note: ^1^ Fish meal: Superprime, TASA Fish Product Co., Ltd., Lima, Peru. ^2^ Cottonseed protein concentrated: Xinjiang Jinlan Plant Protein Co., Ltd., Shihezi, China. ^3^ Soybean meal: Qingdao Bohai Agricultural Development Co., Ltd., Qingdao, China. ^4^ Soybean oil: Yihai Kerry Arawana Holdings Co., Ltd., Shanghai, China. ^5^ Fish oil: Coland Feed Co., Ltd., Wuhan, China. ^6^ Corn starch: Jilin COFCO BIO-Chem & Bio-Energy Marketing Co., Ltd., Changchun, China. ^7^ Microcrystalline cellulose: Shandong Liujia Pharmaceutical Excipients Co., Ltd., Jining, China ^8^ Sodium carboxymethylcellulose: Shanghai Ever Bright Enterprise Development Co., Ltd., Shanghai, China. ^9^ Monocalcium phosphate: Sinopharm Chemical Reagent Co., Ltd., Shanghai, China. ^10^ Vitamin and mineral premix: Dabeinong Aquatic Products Technology Co. Ltd., Wuhan, China. Premix composition (mg/kg diet): VC, 10,500 mg; VE, 4000 mg; VB_2_,1800 mg; VB_6_, 800 mg;VB_1_, 600 mg; VA, 105 mg; VD_3_, 27.5 mg; VB_12_, 3.5 mg; VK_3_, 1100 mg; Nicotinamide, 3000 mg; D-Calcium Pantothenate, 2500 mg; Inositol, 4000 mg; D-Biotin, 10 mg; Folic acid, 250 mg; Fe, 3800 mg; Mg, 3500 mg; Zn, 1750 mg; Mn, 1100 mg; Cu, 760 mg; I, 100 mg; Co, 120 mg; Se, 25 mg; Cr_2_O_3_, 5 mg. ^11^ Choline chloride: Guangdong Nutriera Group, Guangzhou, China. ^12^ Mangiferin: Shanghai Yuanye Bio-Technology Co., Ltd., Shanghai, China. ^13^ Nitrogen free extract contents were calculated by the following formula: Nitrogen free extract content (%) = 100 (%) − moisture (%) − crude protein (%) − crude lipid (%) − ash (%) − crude fiber (%).

**Table 2:** Channel catfish’s primers used for the gene expression assay by qPCR.

Target Genes	Forward (5′-3′)	Reverse (5′-3′)	Accession Number
*sqle*	GTGTAGTGCTCAACGATGTCC	AGCAGGCTCTTCTTAACTGGT	XM_017453059.3
*hmgcr*	CATCTTCTTTGAGCAGGTGGA	GGGTCACTTCCTTCCTGTAAC	XM_017451441.3
*srebf2*	ACGGCGGAGGATTTATAATGG	GGGTCGTCAAACAGATCAGAA	XM_053684143.1
*sp1*	TGTCCACATCAGAGTGTGTCA	TGGATGTTCCTTGAAGGAGCT	XM_017450095.3
*ldlr*	CTGGGTGGACTCTAAACTGCAT	GGAGATGTAAGGTGTTCTGCCA	XM_017492528.3
*abca1*	TTACTGGTGCGGAGAGAAATG	GATTCTCGATGTAGACACCGG	XM_017460992.1
*cyp7a1*	CATCTTCGCGTTCTGCTACAA	CTGTGGCCACTCTTGAATACG	XM_053687284.1
*β-actin*	GGATCTGTATGCCAACACTGT	CAGGTGGGGCAATGATCTTAA	XM_017454668.3
*ef1α*	CACTCCTGGCCTATACGCTG	CAAATGCCACGGTTTCAGGG	XM_053679746.1

Note: *sqle*, squalene epoxidase; *hmgcr*, 3-hydroxy-3-methylglutaryl-CoA reductase; *srebp2*, sterol regulatory element binding transcription factor 2; *sp1*, sp1 transcription factor; *ldlr*, low-density lipoprotein receptor; *abca1*, ATP binding cassette subfamily A member 1; *cyp7a1*, cytochrome P450 family 7 subfamily A member 1; *ef1α*, elongation factor 1 alpha.

**Table 3:** NCTC 1469 cell’s primers used for the gene expression assay by qPCR.

Target Genes	Forward (5′-3′)	Reverse (5′-3′)	Accession Number
sqle	CGGATATTCTCTCTGCTTTGC	AGCTGCTCCTGTTAATGTCGT	NM_009270.3
hmgcr	CAGAGAAAGGTGCAAAGTTCC	CACACCACGTTCATGAGTTTC	XM_036157854.1
β-actin	ACGTTGACATCCGTAAAGACC	ATCGTACTCCTGCTTGCTGAT	NM_007393.5
ef1α	CATGCTCTTCTGGCTTACACC	AGGTGCTGACTTCCTTAACGA	NM_010106.2

Note: sqle, squalene epoxidase; hmgcr, 3-hydroxy-3-methylglutaryl-CoA reductase; ef1α, elongation factor 1 alpha.

## Data Availability

The data presented in this study are available in the article.
